# Assessment of maternal services in China based on WHO’s comprehensive evaluation model

**DOI:** 10.1186/s12913-022-08836-z

**Published:** 2022-12-22

**Authors:** Yalan Liu, Li Yan, Yulin Xia

**Affiliations:** 1 Department of Nosocomial Infection control, Chongqing Public Health Medical Center, Chongqing, 400036 China; 2 Department of quality control, Chongqing University Three Gorges Hospital/Chongqing Three Gorges Central Hospital, Chongqing, China

**Keywords:** Maternal services, Comprehensive evaluation, Rank sum ratio; China

## Abstract

**Background:**

To understand the trend of equalization in maternal services and to guide policy-makers regarding resource allocation and public health policy in China.

**Methods:**

Twelve indicators, including maternal services needs, utilization, and resource allocation, were collected from China Health Statistical Year Book 2010 and 2020. WHO’s comprehensive evaluation model and the non-integral Rank Sum Ratio (RSR) method were used to analyze, rank, and categorize maternal services of 31 provinces (cities, autonomous regions) in China.

**Results:**

All provinces (cities, autonomous regions) are grouped into relative balance areas, low input areas, resource shortage areas, overutilization areas, and resource waste areas. In 2019, there were 18 provinces (cities, autonomous regions) in the relative balanced area, and more than one-half had achieved equal development. Compared to 2009, the resource shortage area decreased from three to zero, and the resource waste area increased from four to six. Among the provinces (cities, autonomous regions) with a type change compared with 2009, eight changed to a relative balance areas, and four showed an improvement.

**Conclusion:**

Under the policy guidance of promoting the equalization of public health services, maternal services are gradually realized. However, several provinces (cities, autonomous regions) still have problems such as the mismatch between resource input and health needs, resource waste, over-utilization, etc. Therefore, specific policies should be formulated according to the actual types to promote the transformation into equalization regions.

## Background

Maternal health is decisive for the country, and maternal mortality is one of the most important indicators to measure the development of social and economic, and social equity [1]. Since 2000, 189 heads of state, including China, signed the Millennium Declaration and committing themselves to achieve target 5, which was to the reduce maternal mortality ratio by three-quarters between 1990 and 2015 [2]. Without exception, all had made continuous efforts to achieve the goal. In China, it had promulgated laws and policies such as the law of the People Republic of China on Maternal and Infant Health Care, the Program for the Development of Chinese Women (2001–2010), and implemented the project of reducing maternal mortality and eliminating neonatal tetanus in 378 counties of 12 provinces in western China, and so on [3, 4]. Under the efforts of the government and people, the target for Millennium Development Goal 5 achieved ahead of schedule in 2014. The past two decades, China has pushed down the maternal mortality ratio at an annualized rate of 6.5% per year, one of the fastest decreases in the world [5, 6]. In recent years, the maternal mortality rate showes a decreasing trend, but there are still gaps among regions and have reached the bottleneck stage [7–9]. The healthy China Initiative 2019 to 2030 states that the maternal mortality ratio should reduced to 12.0 per 100,000 by 2030 [10]. It is a great challenge to take measures to reduce the maternal mortality rate continuously.

In 1996, World Health Organization (WHO) and Swedish International Development Cooperation Agency (SIDA) proposed that equity in health services means that all members of society have the same access to health services, without differences based on social privileges [11]. Therefore, the same access to health services in the same state of health and disease is a fundamental right of members of society. The research showed that the frequency of prenatal check-ups, the proportion of systematic management of high-risk women, the rate of a new delivery, and hospital delivery were the influencing factors of maternal death [12–14]. Strengthening the management of maternal to improve the quality and level of maternal health care is an important measure to implement the Healthy China Strategy. At the same time, the impact of economics on maternal mortality is also crucial [15, 16]. Inadequate funding, resources, and level of health services can lead to poor access to health services, unreasonable allocation, and under-utilization of resources, which directly affect maternal health and make it unequal across regions. Therefore, it is urgent to solve the problem of whether the decrease in maternal mortality is caused by insufficient investment or inadequate utilization of resources, and whether health needs and health supply are balanced.

In 2009, promoting equalization of Basic Public Health services was one of the five major tasks of China’s health care reform, aiming to ensure that all residents in urban and rural areas could access the services and focus on equity [17, 18]. The seventh population census in 2020 showed that population growth has slowed, mainly due to the continuous decrease in fertile women that has led to a weakening of the momentum of population growth and a slight decline in the fertility level. Meanwhile, the proportion aged 65 and above reached 13.50%, which was higher than the world average of 9.3% [19]. With the aging population increasing and fertility level decreasing, it has put forward higher requirements for the health of fertile women, especially for maternal. Maternal health is the foundation of universal health and is the main object of public health services. Equity is the focus of research.

The study aimed to understand the relationship among maternal service needs, utilization, and resource allocation of 31 provinces (cities, autonomous regions) in China in 2009 and 2019, using a multi-index comprehensive evaluation method. Also, from the perspective of equity and efficiency to understand the changing trends and differences in the region, and provide guidance for policymakers regarding resource allocation and public health policy.

## Methods

### Data sources

The data that included maternal services needs, utilization, and resource allocation was retrieved from the China Health Statistics Yearbook 2010 and 2020 (http://www.nhc.gov.cn/mohwsbwstjxxzx/tjtjnj/new_list.shtml) [20, 21]. Twelve indicators were selected to comprehensively evaluate and analyze the equalization of maternal services across 31 provinces (cities, autonomous regions).

The three maternal services needs indicators X_1_ ~ X_3_ are maternal mortality rate (1/100,000), the proportion of obstetric hemorrhage in maternal deaths (%), and pregnancy-induced hypertension.

in maternal deaths(%). The five indicators X_4_ ~ X_8_ in the utilization of maternal services are.

registration rate (%), system management rate (%), prenatal checkup rate (%), postpartum visitrate (%), and hospital delivery rate (%). The maternal system management rate refers to the.

number of women who have received early pregnancy checkups, antenatal checkups, sterile.

delivery, and postpartum visits within 28 days after delivery to the number of live births in a given area during the year. Four resource allocation indicators X_9_ ~ X_12_ are the number of medical institutions (1/100,000), the number of practicing (assistant) physicians (1/1000), and the number of registered nurses (1/1000), the number of obstetrics and gynecology beds (1/100,000). Combined with the responsibilities of maternal health care and the desirability of indicators, the institutions includecomprehensive hospitals, primary medical institutions, maternal and child health hospitals (stations). Health technicians are involved physicians and nurses in maternal and child health hospitals (stations).

### The RSR method

The fundamental theory of the RSR method is that a dimensionless statistical indicator is calculated from an n × m matrix using rank conversion. After this calculation, the distribution of RSR using parametric statistical methods. Generally, the RSR indicator ranges from zero (worst) to one (best) and follows a normal distribution. Additionally, the status (worst/best) uses the RSR order or a set of ordinal classifications to evalute [22].

In our study, the RSR method was used to rank and classify 31 provinces in China in terms of maternal service needs, utilization, and resource allocation, respectively. All indicators used the same weight, and considering that the integer rank method would lose the original data information, the non-integer rank RSR was adopted to overcome the disadvantage of losing the quantitative information of the original data.

The detailed processes are as follows:

1. Rank the indicators of maternal services in each province, the high-quality ranked in ascending,and the low-quality indicator ranked in descending order, such as

$${R}_{high- quality}=1+\frac{\left(n-1\right)\times \left(X-{X}_{\textrm{min}}\right)}{X_{\textrm{max}}-{X}_{\textrm{min}}}$$

$${R}_{low- quality}=1+\frac{\left(n-1\right)\times \left({X}_{\textrm{max}}-{X}_{\textrm{min}}\right)}{X_{\textrm{max}}-{X}_{\textrm{min}}}$$

Where R is the rank of each maternal services indicator of 31 provinces in China, X is the original value, *n* = 31, Except for maternal mortality rate, the other is high quality in the study.

2. Calculate the value of RSR, the equation as follows

$$RSR=\frac{1}{m\times n}\sum \limits_{j=1}^i{R}_{ij}$$

Where *R*_ij_ is the rank of indicators in maternal services needs, utilization, and resource allocation, m is the number of indicator in each dimension, n is the number of provinces, i = 1,2∙∙∙n, j = 1,2∙∙∙m.

3. Determine the distribution of RSR. Sort the RSR from small to large; calculate the downward

cumulative frequency P (average rank/n*100%) according to the cumulative frequency and average rank; then convert it into Probit.

4. Calculate the regression equation. The value of RSR as the dependent variable and Probit as the

independent variable to fit the linear regression equations of maternal services needs, utilization, and resource allocation, then calculate the fitted RSR of each region.

$$\overset{\wedge }{RSR}=a+b\times probit$$

5. Grading and sorting. Concerning the commonly used three-grading table, the Probit critical values are substituted into the regression equation to calculate the RSR critical values for grading, and variance analysis is used to compare the differences among groups. SNK-q is used for pairwise comparison; the statistically significant level is set at *P* < 0.05

### WHO’s comprehensive evaluation model

Based on the investigation of health services in many countries and regions, the WHO proposed to combine health service needs, utilization, and resource input, and grade the sample mean of the three categories of indicators to form eight evaluation types from A to H [23], as shown in Table 1.

**Table 1:** WHO’s comprehensive evaluation model of health service

Utilization	High needs		Low needs	
	High resources	Low resources	High resources	Low resources
High	A	B	E	F
Low	C	D	G	H

Type A and H indicate appropriate allocation of resources. The high resource utilization is types B and F, types C and E mean low and over resource utilization, respectively. Types D and G indicate the low and over the investment of resources.

In China, scholar Chen H and others established the comprehensive evaluation modelof Basic Public Health services, and combined the method of RSR, which is grouped into five categories, including relative balance area (types A,H, and F), low input area (type B), resource shortage area (type D), overutilization area (type E), and resource waste area (types C and G), which is detailed in Table 2 [24].

**Table 2:** The comprehensive evaluation model of basic public health services in China

Utilization	High needs			Medium needs			Low needs		
	High resource	Medium resource	Low resource	High resource	Medium resource	Low resource	High resources	Medium resource	Low resource
High	A	B	B	E	F	B	E	E	F
Medium	C	D	B	G	A	B	G	E	F
Low	C	D	D	G	C	D	G	G	H

In our study, we used it for reference to carry out comprehensive evaluation of maternal services.

## Results

### The RSR value in needs, utilization, and resource allocation of maternal services across China in 2009 and 2019

According to the RSR value ranking evaluation of the maternal services in each region, in 2009, the top three maternal health needs were in Xizang, Qinghai, and Xinjiang. The utilization of maternal health care was in Beijing, Zhejiang, and Shandong, and the resource allocations were in Beijing, Ningxia, and Xinjiang. While ten years later, the top three maternal health needs were in Xizang, Tianjin, and Qinghai; maternal health care utilization was in Zhejiang,Tianjin, and Guangxi; resource allocations were in Guizhou, Ningxia, and Qinghai (Table 3).

**Table 3:** The RSR value in needs, utilization and resource allocation of maternal services across China in 2009 and 2019

Region	Needs		Utilization		Resource allocation	
	2009	2019	2009	2019	2009	2019
	RSR(rank)	RSR(rank)	RSR(rank)	RSR(rank)	RSR(rank)	RSR(rank)
Beijing	0.147(29)	0.032(31)	0.814(1)	0.866(19)	0.557(1)	0.382(16)
Tianjin	0.175(28)	0.519(2)	0.736(12)	0.973(2)	0.348(14)	0.069(31)
Hebei	0.290(13)	0.205(25)	0.705(15)	0.856(22)	0.360(13)	0.377(18)
Shanxi	0.240(21)	0.272(15)	0.578(26)	0.793(27)	0.496(5)	0.331(23)
Inner Mongolia	0.253(16)	0.209(24)	0.743(11)	0.930(7)	0.503(4)	0.492(7)
Liaoning	0.214(25)	0.321(7)	0.796(4)	0.904(14)	0.227(26)	0.156(29)
Jilin	0.245(19)	0.303(10)	0.591(25)	0.937(6)	0.441(10)	0.231(25)
Heilongjiang	0.305(10)	0.342(5)	0.691(17)	0.913(12)	0.312(19)	0.199(28)
Shanghai	0.355(8)	0.033(30)	0.616(23)	0.962(4)	0.252(24)	0.091(30)
Jiangsu	0.229(22)	0.248(20)	0.785(6)	0.846(24)	0.099(31)	0.222(26)
Zhejiang	0.146(30)	0.276(14)	0.804(2)	0.984(1)	0.490(6)	0.493(6)
Anhui	0.304(11)	0.250(19)	0.290(30)	0.857(21)	0.117(30)	0.211(27)
Fujian	0.144(31)	0.268(16)	0.727(13)	0.899(16)	0.253(23)	0.337(22)
Jiangxi	0.254(15)	0.255(17)	0.658(19)	0.915(10)	0.327(16)	0.370(19)
Shandong	0.229(23)	0.240(22)	0.803(3)	0.890(17)	0.301(21)	0.412(14)
Henan	0.245(18)	0.276(13)	0.780(8)	0.746(29)	0.304(20)	0.380(17)
Hubei	0.262(14)	0.252(18)	0.751(10)	0.916(9)	0.323(17)	0.424(13)
Hunan	0.208(26)	0.201(26)	0.705(16)	0.939(5)	0.313(18)	0.492(8)
Guangdong	0.253(17)	0.170(28)	0.722(14)	0.907(13)	0.447(8)	0.444(12)
Guangxi	0.218(24)	0.218(23)	0.755(9)	0.970(3)	0.442(9)	0.526(5)
Hainan	0.320(9)	0.129(29)	0.506(29)	0.852(23)	0.396(12)	0.387(15)
Chongqing	0.425(4)	0.183(27)	0.591(24)	0.903(15)	0.160(28)	0.261(24)
Sichuan	0.358(7)	0.310(9)	0.630(21)	0.913(11)	0.199(27)	0.349(21)
Guizhou	0.384(5)	0.290(11)	0.578(27)	0.865(20)	0.154(29)	0.569(1)
Yunnan	0.375(6)	0.325(6)	0.683(18)	0.753(28)	0.241(25)	0.466(9)
Xizang	0.863(1)	0.785(1)	0.032(31)	0.032(31)	0.474(7)	0.531(4)
Shanxi	0.201(27)	0.244(21)	0.782(7)	0.921(8)	0.415(11)	0.462(10)
Gansu	0.299(12)	0.284(12)	0.656(20)	0.887(18)	0.331(15)	0.451(11)
Qinghai	0.640(2)	0.439(3)	0.539(28)	0.808(26)	0.300(22)	0.532(3)
Ningxia	0.241(20)	0.386(4)	0.791(5)	0.723(30)	0.538(2)	0.547(2)
Xinjiang	0.473(3)	0.313(8)	0.628(22)	0.821(25)	0.512(3)	0.364(20)

### The distribution of RSR in needs, utilization, and resource allocation of maternal services across China in 2009 and 2019

According to the value of RSR and Probit (Table 4), six regression equations were obtained. In 2009, regression equations in needs, utilization, and resource allocation of maternal services were RSR_1_= 0.132Probit-0.37 (*r* = 0.798), RSR_2_ = 0.136Probit-0.029 (*r* = 0.693), RSR_3_ = 0.125Probit-0.293 (*r* = 0.964), respectively. In 2019, they were RSR_4_ = 0.126Probit-0.36 (*r* = 0.849), RSR_5_ = 0.113Probit + 0.281 (*r* = 0.444), RSR_6_ = 0.130Probit-0.289 (*r* = 0.921). The results of variance analysis showed that all regression equations were statistically significant (*P* < 0.05).

**Table 4:** The distribution of RSR in needs, utilization and resource allocation of maternal services across China in 2009 and 2019

Region	Needs				Utilization				Resources allocation			
	2009		2019		2009		2019		2009		2019	
	*P*	Probit	*P*	Probit	*P*	Probit	*P*	Probit	*P*	Probit	*P*	Probit
Beijing	9.7	3.700	3.2	3.151	99.2	7.406	41.9	4.796	99.2	7.406	51.6	5.040
Tianjin	12.9	3.869	96.8	6.849	64.5	5.372	96.8	6.849	58.1	5.204	3.2	3.151
Hebei	61.3	5.287	22.6	4.247	54.8	5.122	32.3	4.540	61.3	5.287	45.2	4.878
Shanxi	35.5	4.628	54.8	5.122	19.4	4.135	16.1	4.011	87.1	6.131	29.0	4.448
Inner Mongolia	48.4	4.960	25.8	4.351	67.7	5.460	80.6	5.865	90.3	6.300	77.4	5.753
Liaoning	22.6	4.247	80.6	5.865	90.3	6.300	58.1	5.204	19.4	4.135	9.7	3.700
Jilin	41.9	4.796	71.0	5.552	22.6	4.247	83.9	5.989	71.0	5.552	22.6	4.247
Heilongjiang	71.0	5.552	87.1	6.131	48.4	4.960	64.5	5.372	41.9	4.796	12.9	3.869
Shanghai	77.4	5.753	6.5	3.482	29.0	4.448	90.3	6.300	25.8	4.351	6.5	3.482
Jiangsu	29.0	4.448	38.7	4.713	83.9	5.989	25.8	4.351	3.2	3.151	19.4	4.135
Zhejiang	6.5	3.482	58.1	5.204	96.8	6.849	99.2	7.406	83.9	5.989	83.9	5.989
Anhui	67.7	5.460	41.9	4.796	6.5	3.482	35.5	4.628	6.5	3.482	16.1	4.011
Fujian	3.2	3.151	51.6	5.040	61.3	5.287	51.6	5.040	29.0	4.448	32.3	4.540
Jiangxi	54.8	5.122	48.4	4.960	41.9	4.796	71.0	5.552	51.6	5.040	41.9	4.796
Shandong	32.3	4.540	32.3	4.540	93.5	6.518	48.4	4.960	35.5	4.628	58.1	5.204
Henan	45.2	4.878	61.3	5.287	77.4	5.753	9.7	3.700	38.7	4.713	48.4	4.960
Hubei	58.1	5.204	45.2	4.878	71.0	5.552	74.2	5.649	48.4	4.960	61.3	5.287
Hunan	19.4	4.135	19.4	4.135	51.6	5.040	87.1	6.131	45.2	4.878	80.6	5.865
Guangdong	51.6	5.040	12.9	3.869	58.1	5.204	61.3	5.287	77.4	5.753	64.5	5.372
Guangxi	25.8	4.351	29.0	4.448	74.2	5.649	93.5	6.518	74.2	5.649	87.1	6.131
Hainan	74.2	5.649	9.7	3.700	9.7	3.700	29.0	4.448	64.5	5.372	54.8	5.122
Chongqing	90.3	6.300	16.1	4.011	25.8	4.351	54.8	5.122	12.9	3.869	25.8	4.351
Sichuan	80.6	5.865	74.2	5.649	35.5	4.628	67.7	5.460	16.1	4.011	35.5	4.628
Guizhou	87.1	6.131	67.7	5.460	16.1	4.011	38.7	4.713	9.7	3.700	99.2	7.406
Yunnan	83.9	5.989	83.9	5.989	45.2	4.878	12.9	3.869	22.6	4.247	74.2	5.649
Xizang	99.2	7.406	99.2	7.406	3.2	3.151	3.2	3.151	80.6	5.865	90.3	6.300
Shanxi	16.1	4.011	35.5	4.628	80.6	5.865	77.4	5.753	67.7	5.460	71.0	5.552
Gansu	64.5	5.372	64.5	5.372	38.7	4.713	45.2	4.878	54.8	5.122	67.7	5.460
Qinghai	96.8	6.849	93.5	6.518	12.9	3.869	19.4	4.135	32.3	4.54	93.5	6.518
Ningxia	38.7	4.713	90.3	6.300	87.1	6.131	6.5	3.482	96.8	6.849	96.8	6.849
Xinjiang	93.5	6.518	77.4	5.753	32.3	4.540	22.6	4.247	93.5	6.518	38.7	4.713

### Grading and sorting maternal services needs, utilization, and resource allocation across China in 2009 and 2019

The Probit critical values of 4 and 6 were substituted into all regression equations to calculate the RSR as the basis of classification, and then calculated the fitted values of RSR in maternal service needs, utilization, and resource allocation of all provinces, and the final results were shown in Table 5 and Table 6.

**Table 5:** Grading and sorting maternal services needs, utilization and resource allocation across China in 2009

Level	Probit	Needs			Utilization			Resources allocation		
		RSR	Fitted value of RSR		RSR	Fitted value of RSR		RSR	Fitted value of RSR	
Low	≤4	≤ 0.158	Fujian	0.046	≤ 0.514	Xizang	0.399	≤ 0.208	Jiangsu	0.102
			Zhejiang	0.089		Anhui	0.444		Guizhou	0.170
			Beijing	0.118		Hainan	0.473		Anhui	0.143
			Tianjin	0.140		Qinghai	0.496		Chongqing	0.192
Medium	4^~^	0.158 ~	Shanxi	0.159	0.514 ~	Guizhou	0.515	0.208 ~	Sichuan	0.209
			Hunan	0.175		Shanxi	0.532		Liaoning	0.225
			Liaoning	0.190		Jilin	0.548		Yunnan	0.239
			Guangxi	0.204		Chongqing	0.562		Shanghai	0.252
			Shandong	0.217		Shanghai	0.575		Fujian	0.264
			Jiangsu	0.229		Xinjiang	0.587		Qinghai	0.276
			Shanxi	0.240		Sichuan	0.599		Shandong	0.287
			Ningxia	0.252		Gansu	0.611		Henan	0.297
			Jilin	0.263		Jiangxi	0.622		Heilongjiang	0.308
			Henan	0.274		Yunnan	0.633		Hunan	0.318
			Guangdong	0.284		Heilongjiang	0.644		Hubei	0.328
			Inner Mongolia	0.295		Hunan	0.655		Jiangxi	0.338
			Jiangxi	0.306		Hebei	0.666		Gansu	0.349
			Hubei	0.316		Guangdong	0.677		Tianjin	0.359
			Hebei	0.327		Fujian	0.689		Hebei	0.369
			Gansu	0.339		Tianjin	0.700		Hainan	0.380
			Anhui	0.350		Inner Mongolia	0.712		Shanxi	0.391
			Heilongjiang	0.362		Hubei	0.725		Jilin	0.402
			Hainan	0.375		Guangxi	0.738		Guangix	0.415
			Shanghai	0.389		Henan	0.752		Shandong	0.428
			Sichuan	0.404		Shanxi	0.767		Xizang	0.442
			Yunnan	0.420		Jiangsu	0.784		Zhejiang	0.457
High	≥6	≥ 0.422	Guizhou	0.439	≥ 0.785	Ningxia	0.803	≥ 0.458	Shanxi	0.475
			Chongqing	0.461		Liaoning	0.826		Inner Mongolia	0.496
			Xinjiang	0.490		Shandong	0.856		Xinjiang	0.523
			Qinghai	0.534		Zhejiang	0.901		Ningxia	0.565
			Xizang	0.607		Beijing	0.976		Beijing	0.635

**Table 6:** Grading and sorting maternal services needs, utilization and resource allocation across China in 2019

Level	Probit	Needs			Utilization			Resources allocation		
		RSR	Fitted value of RSR		RSR	Fitted value of RSR		RSR	Fitted value of RSR	
Low	≤4	≤ 0.142	Beijing	0.035	≤ 0.732	Xizang	0.637	≤ 0.232	Tianjin	0.122
			Shanghai	0.077		Ningxia	0.674		Shanghai	0.165
			Hainan	0.104		Henan	0.699		Liaoning	0.193
			Guangdong	0.125		Yunnan	0.718		Heilongjiang	0.215
Medium	4^~^	0.142 ~	Chongqing	0.143	0.732 ~	Shanxi	0.734	0.232 ~	Anhui	0.234
			Hunan	0.159		Qinghai	0.748		Jiangsu	0.250
			Hebei	0.173		Xinjiang	0.760		Jilin	0.265
			Inner Mongolia	0.186		Jiangsu	0.772		Chongqing	0.278
			Guangxi	0.198		Hainan	0.783		Shanxi	0.291
			Shandong	0.209		Hebei	0.793		Fujian	0.303
			Shanxi	0.220		Anhui	0.803		Sichuan	0.314
			Jiangsu	0.231		Guizhou	0.813		Xinjiang	0.325
			Anhui	0.241		Beijing	0.822		Jiangxi	0.336
			Hubei	0.252		Gansu	0.832		Hebei	0.347
			Jiangxi	0.262		Shandong	0.841		Henan	0.357
			Fujian	0.272		Fujian	0.850		Beijing	0.368
			Shanxi	0.282		Chongqing	0.859		Hainan	0.379
			Zhejiang	0.292		Liaoning	0.868		Shandong	0.389
			Henan	0.303		Guangdong	0.878		Hubei	0.400
			Gansu	0.314		Heilongjiang	0.888		Guangdong	0.411
			Guizhou	0.325		Sichuan	0.897		Gansu	0.423
			Jilin	0.336		Jiangxi	0.908		Shanxi	0.435
			Sichuan	0.348		Hubei	0.919		Yunnan	0.447
			Xinjiang	0.361		Shanxi	0.930		Hunan	0.461
			Liaoning	0.375		Inner Mongolia	0.943		Inner Mongolia	0.475
			Yunnan	0.391		Jilin	0.957		Zhejiang	0.492
High	≥6	≥ 0.392	Heilongjiang	0.409	≥ 0.958	Hunan	0.973	≥ 0.493	Guangxi	0.510
			Ningxia	0.43		Shanghai	0.992		Xizang	0.532
			Qinghai	0.457		Guangxi	1.017		Qinghai	0.561
			Tianjin	0.499		Tianjin	1.054		Ningxia	0.604
			Xizang	0.569		Zhejiang	1.117		Guizhou	0.676

The test of variance consistency showed no significant differences among groups in each dimension (*P* > 0.05). In 2009, an analysis of variance showed that the statistics value F of maternal service needs, utilization, and resource allocation were 36.424, 36.431, and 36.363, respectively, and 36.565, 36.306, and 36.507. In 2019, there were significant differences in the fitted values of RSR among groups of each dimension (*P* < 0.05). The SNK-q test showed a significant difference pairwise, and the grading was reasonable.

### The comprehensive evaluation of maternal services in 2009 and 2019

According to the results of grading, all regions were filled into the model (Table 7). In 2009, the relative balance area, low input area, resource shortage area, resource waste area, and overutilization area were16, 3, 4, 3, and 5, respectively. In 2019, they were 18, 3, 6, 0, and 4, respectively.

**Table 7:** The comprehensive evaluation of maternal services in 2009 and 2019

Year	Utilization	High needs			Medium needs			Low needs		
		High resources	Medium resources	Low resources	High resources	Medium resources	Low resources	High resources	Medium resources	Low resources
2009	High				Ningxia	Liaoning,Shandong		Beijing	Zhejiang	
	Medium	Xinjiang		Guizhou,Chongqing	Shanxi,Inner Mongolia	Shanxi,Hunan,Guangxi,Jilin,Henan,Guangdong,Jiangxi,Hubei,Hebei,Shanghai,Heilongjiang,Gansu,Sichuan,Yunnan	Jiangsu		Fujian,Tianjin	
	Low		Qinghai,Xizang			Hainan	Anhui			
2019	High			Tianjin	Guangxi	Hunan,Zhejiang				Shanghai
	Medium	Qinghai		Heilongjiang	Guizhou	Chongqing,Hebei,Shanxi,Xinjiang,Hubei,Shanxi,Shandong,Fujian,Jiangsu,Anhui,Jiangxi,Gansu,Jilin,Sichuan,Inner Mongolia	Liaoning		Beijing,Hainan,Guangdong	
	Low	Xizang,Ningxia				Henan,Yunnan				

## Discussion

Since China announced a health reform blueprint to achieve universal coverage by 2020, the equalization of public health services between different regional, urban and rural areas has gradually improved [25–29]. The research showed that 18 regions were in the relative balance area in maternal health resources allocation and health needs, and more than half had met the requirements for equitable development, which was higher than that of 16 provinces in 2009. In addition, the overutilization area decreased one province, and the resource shortage area decreased three. Compared to 2009, several regions experienced a type shift, eight changed from non-balanced to a relative balance area, and four showed an improvement in type. It indicated that under the guidance of the Public Health Service policy, including system construction, project promotion, serviceability strength, and the fund guarantee, the equalization of maternal services were gradually being realized.

In 2019, three regions were still in the low input area, and the resource allocation was not meet the needs. From the perspective of regional GDP, these were at the bottom in 2019. The economy could limit fund-raising ability, which leads to insufficient investment in infrastructures such as institutions, personnel, and beds; the RSR value of resource allocation ranked in the bottom five. At the same time, the limit on salary, training, and promotion opportunities resulted in brain drain, personnel shortage, insufficient service capacity, and other issues. According to research statistics, the full-time public health personnel in primary medical health institutions accounted for only 10% of health technicians in 2017, and 31% of physicians in urban community health service centers (stations) failed to meet the requirements of practicing assistant physicians in 2015 [30]. Therefore, it is critical to strengthen financial investment and consolidate the appropriation of special funds.

Ten years later, 10 regions still with low efficiency in maternal services, of which six were resource waste and four were over-utilized areas, resource waste coexists with overutilization. Under the guidance of macro policies, while the whole country was actively implementing the public health services tasks, the control of the efficiency of resource utilization had been neglected, especially for consideration of professional public health institutions. Therefore, it is urgent to integrate resources and optimize the utilization efficiency of health resources. We should combine the system of grading diagnosis and treatment to improve the capacity of primary-level medical institutions, promote the development of appropriate health technologies, and give full play to the role of primary-level medical institutions. Therefore, it is urgent to integrate and optimize the efficiency of resources. The hierarchical diagnosis and treatment and medical association should combine to improve the ability of maternal health, promote the development of appropriate health technology, and give full play to the role of primary medical institutions. Resource input was higher than health needs in four regions, which lead to oversupply, so the cost measurement mechanism should be improved to ensure that resources such as personnel and beds are set up according to demand.

## Conclusions

Based on the WHO’s comprehensive evaluation model of Health Services, this study evaluated the needs and supply of maternal services from the perspective of equity and efficiency in China. The results showed that the equalization of maternal services was gradually being realized under the guidance of the equalization of Public Health Service policies. However, there were still some problems, such as the mismatch of resource input, health needs and waste, and the over-utilization of resources. So the non-balanced regions should formulate targeted policies in the light of their specific circumstances to promote their transformation to equalization. However, due to the availability of data, it is unable to include all technicians serving maternal, and unable to obtain dedicated public health funding. It is suggested the relevant departments should refine the statistical classification of information as much as possible and make more public resources available.

## Data Availability

The data that support the findings of this study are available from the first author upon reasonable request.
